# The mechanism of coupling and coordination between the rule of law and sustainable development from a system theory perspective: evidence from China

**DOI:** 10.3389/fpubh.2025.1597761

**Published:** 2025-05-22

**Authors:** Qiancheng Zhou, Liwei Zhang, Chenxi Nian

**Affiliations:** ^1^School of Public Administration, Jilin University, Changchun, China; ^2^School of Law, Central China Normal University, Wuhan, China

**Keywords:** rule of law, sustainable development, coupling coordination mechanism, the system theory, quantitative analysis

## Abstract

The rule of law and sustainable development are two core elements in addressing the challenges of national reform and transformation. To further clarify the roles and relationship between these two aspects, this paper innovatively proposes a coupling coordination mechanism for the rule of law and sustainable development. Using evidence from China, this study first constructs an indicator system for both elements from a systems theory perspective. Through empirical research, the entropy method and coupling coordination degree model are employed to measure the development levels and coupling coordination effects of the rule of law and sustainable development. Additionally, the grey relational model is utilized to quantify the factors influencing the interaction between the two during the coupling process, and practical pathways for promoting their coordinated development are proposed. The findings reveal a significant upward trend in the coupling coordination relationship between the rule of law and sustainable development over the past decade, evolving from severe imbalance to high-quality coordination. Notably, the rule of law environment and social rule of law have a significant impact on sustainable development, while economic, ecological, and political sustainability notably influence the rule of law. This study not only elucidates the coupling coordination relationship and mutual influences between the rule of law and sustainable development but also provides theoretical insights and practical guidance for advancing national modernization and addressing development challenges in other countries.

## 1 Introduction

Currently, the world is undergoing unprecedented changes not seen in a century, and the relationship between social stability and sustainable development is attracting increasing attention. China, as the largest developing country and one of the most populous nations in the world, faces the challenge of promoting sustainable and healthy national development while ensuring that the benefits of development are more widely shared among ordinary citizens (1). With the rapid economic growth, Chinese social structure and interest patterns have undergone profound changes. At present, China is at a critical juncture of development transformation and upgrading, facing issues such as intensified resource constraints, worsening environmental pollution, prominent social conflicts, widening wealth gaps, and unbalanced and insufficient development (2, 3). These challenges not only affect social harmony and stability but also constrain the sustained and healthy development of the economy, threatening the quality of life and well-being of the populace (4). They have become obstacles that must be overcome on the path to achieving the goal of building a modernized nation. Therefore, managing the relationship between social stability and sustainable development is not only an urgent task for national development but also a significant strategic choice related to the long-term development and rejuvenation of the nation (5, 6).

In response to the many obstacles encountered during the development process, the Chinese government has adopted a path of jointly promoting the rule of law and sustainable development (7). On one hand, the rule of law is an important means of advancing social stability and improving social relations. It serves as a fundamental approach to modern state governance, regulating social behavior through legal norms, ensuring social fairness and justice, and maintaining social order and stability (8, 9).

The rule of law is a key lever for the Chinese government in promoting modernization (10). As the largest developing country in the world and a nation that has made significant progress in systematically addressing poverty alleviation, China has continuously strengthened its rule of law and improved its legal framework. This ensures that various laws and regulations comprehensively and effectively protect citizens’ legitimate rights and interests, uphold social fairness and justice, and promote high-level social development. Advancing the rule of law provides clear rules and procedures for social development, enhances the transparency and predictability of governance processes, and thereby improves governance effectiveness (11). On the other hand, sustainable development is a core requirement for Chinese economic development in the new era. It emphasizes the quality and efficiency of development, focuses on innovation-driven and structural optimization, and pursues coordinated development across economic, political, cultural, social, and ecological dimensions (12, 13). Sustainable development is a primary goal of industrial upgrading and economic structural transformation; it not only promotes long-term stable economic growth but also provides a solid material foundation and broader development space for social governance (14).

Coupling and coordinating the rule of law with sustainable development is an effective choice for addressing and solving the complex issues currently faced in development (15). Through the rule of law, a solid institutional guarantee for sustainable development is provided, ensuring policy continuity and stability while achieving fairness and justice in the economic and social development process, thus laying the foundation for long-term healthy economic development (16). Meanwhile, sustainable development can offer a strong material basis and support for the rule of law (17). The economic growth and technological advancements brought about by sustainable development can provide more resources and conditions for the construction of the rule of law, leading to a more comprehensive legal system and strengthened judicial fairness, thereby promoting the continuous optimization and advancement of the rule of law environment (18). This mutually reinforcing and integrating relationship can not only jointly build a coordinated development mechanism for the healthy development of the nation, enhancing the sense of gain, happiness, and security among the populace but also provide strong support and guarantees for achieving the modernization of the national governance system and governance capacity (19, 20), ultimately realizing social harmony, stability, and long-term development (21, 22). Therefore, studying the coupling and coordination relationship between the rule of law and sustainable development is of significant importance (23, 24).

Based on the importance of both the rule of law and sustainable development, this article proposes the following research questions: How can an appropriate indicator system be constructed, and what theoretical foundation should be used to evaluate the rule of law and sustainable development? As the largest developing country in the world, what has been the level of development of the rule of law and sustainable development in China over the past decade, and what trends have emerged in their development levels during these years? What is the relationship between the rule of law and sustainable development? Is there a close coordination relationship between the two, and has a tight coupling coordination mechanism been established? What are the key factors affecting the coupled and coordinated development of both, and how can we promote their coordinated development?

**Figure 1:**
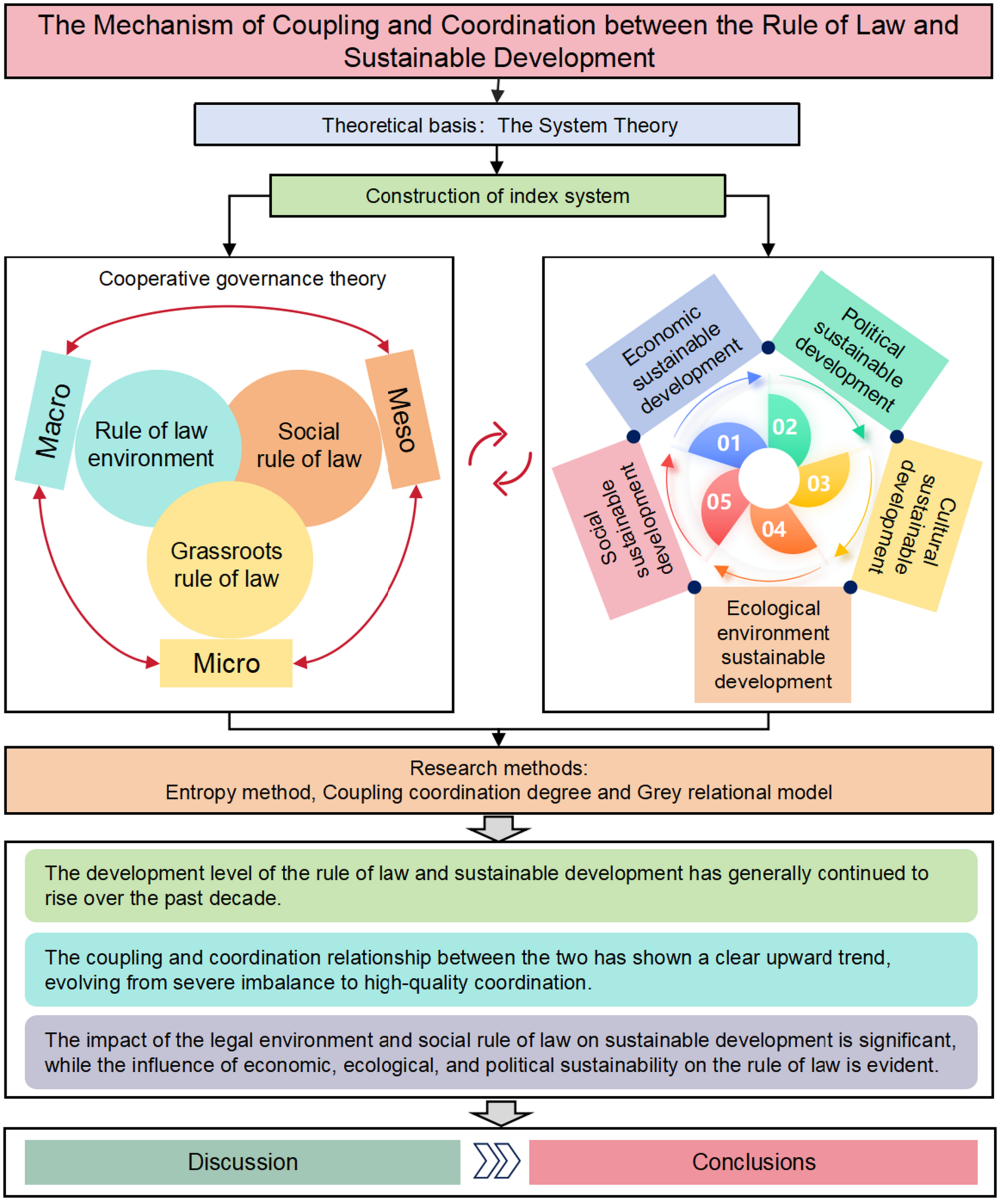
Research framework diagram.

To address these questions, this article first constructs evaluation indicator systems for both the rule of law and sustainable development using systems theory. Then, using the entropy method, it calculates the development levels of both over the past decade. Next, it assesses the coupling and coordination effects between the two using the coupling coordination degree model. Finally, it evaluates the key factors influencing their coupled and coordinated development through grey relational analysis and provides corresponding recommendations. This process will help us gain a more comprehensive understanding of the interactive relationship between the two and how they jointly impact socio-economic development in China. The research framework for this article is illustrated in Figure 1.

## 2 Review of literature and theoretical framework

As an essential component of modern national governance systems, the rule of law has garnered extensive attention from academia in recent years. The rule of law is a macro and multidimensional concept that involves managing and regulating social behavior through legal means to maintain social order, safeguard citizens’ rights, and promote social justice and harmony (25). It refers to governing society through the formulation, implementation, and enforcement of laws. The rule of law signifies that a society implements governance through a series of meticulous legal operational mechanisms, including scientific legislation, strict law enforcement, and fair judicial practices, all of which are interconnected and indispensable, forming a complete chain of rule of law practice (26). In this process, the law transcends mere behavioral norms; as an efficient tool for social governance, it plays a crucial role in leading social progress and ensuring fairness and justice. The rule of law is a governance model widely adopted by countries around the world, both developed and developing, as they actively explore and practice rule of law paths that align with their national conditions, aiming to promote social stability and sustainable development through strengthened rule of law construction (27, 28).

In the context of the new era’s global development wave, sustainable development has become the core strategic orientation and long-term goal of China’s economic development (29). The explicit articulation of this goal not only reflects a profound shift in China’s economic development thinking and strategic direction but also signifies that China is gradually abandoning the traditional model of solely pursuing rapid economic growth (30, 31). Instead, the focus of development is shifting towards enhancing the quality and efficiency of growth, striving to achieve a more robust, lasting, and balanced development trajectory while maintaining steady economic growth. The core of the sustainable development strategy emphasizes and advocates a dual approach of innovation-driven growth and industrial structure optimization to propel the economy towards higher quality, greater efficiency, increased fairness, and enhanced sustainability (32, 33). Sustainable development represents not just a profound transformation within the economic sphere but also manifests across multiple key areas and dimensions, including economic, political, cultural, social, and ecological environments.

**Figure 2:**
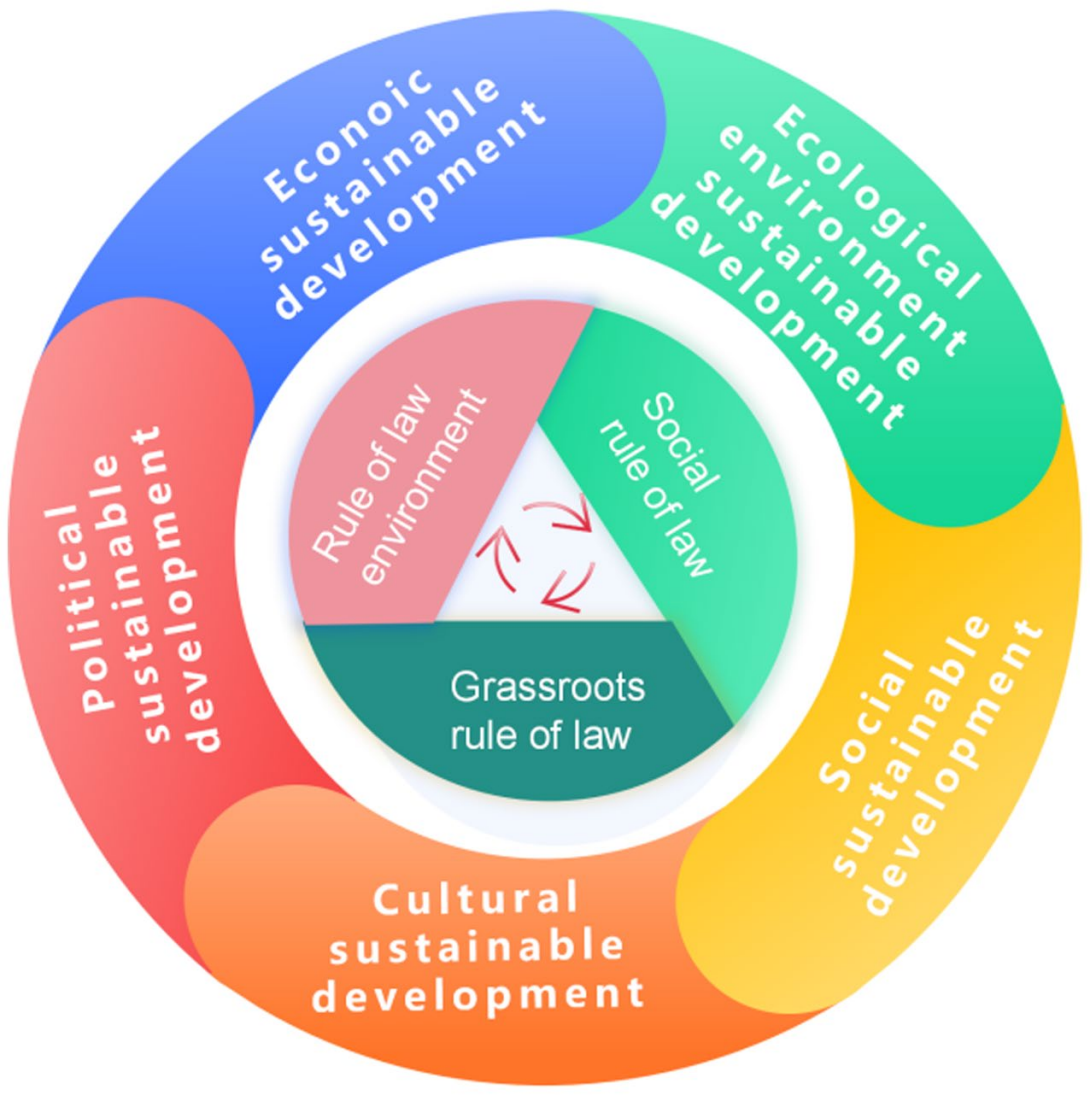
Theoretical framework of the system theory research.

The system theory emphasizes the interconnections and interactions among various entities, providing a powerful analytical tool for understanding complex social phenomena. To better conduct this research, this paper draws on the general systems theory proposed by renowned scholar Qaim (34), using it as the foundation for constructing the research framework (Figure 2). By employing systems theory, this study aims to comprehensively grasp the interactions and influences between the rule of law and sustainable development (35), thereby providing a scientific theoretical basis for the formulation and implementation of relevant policies (36). The research delves into the application of systems theory in the social sciences, particularly how the principles and methods of systems theory can be applied to the seemingly independent yet intrinsically interconnected fields of rule of law and sustainable development (37). We not only focus on the development levels of the rule of law and sustainable development individually but also pay special attention to the dynamic interactions between the two, thereby revealing the intrinsic connections between the rule of law and sustainable development more scientifically (38).

From the perspective of the system theory, the coupling coordination mechanism between the rule of law and sustainable development is viewed as an effective pathway for achieving healthy social development. This mechanism emphasizes the mutual promotion and constraint between the two systems, optimizing resource allocation and structural adjustments to achieve overall system optimization. Through this coupling coordination mechanism, a positive interaction between the rule of law and sustainable development can be realized, providing strong support for building a modern economic system and achieving comprehensive national progress and long-term development.

## 3 Construction of the indicator system

The division of the indicator system aims to comprehensively and objectively reflect the actual situation and development trends in the fields of the rule of law and sustainable development, providing a basis for formulating more scientific and reasonable development strategies (39). First, this paper constructs the indicator system by drawing on previous research to ensure its rationality and comprehensiveness. Second, the design of the indicator system also considers the availability and comparability of data. By selecting representative and quantifiable indicators and standardizing them, data from different fields and time points can be made comparable, allowing for a more accurate assessment of the levels and trends of the rule of law and sustainable development (40).

The classification of indicators in this paper is primarily based on the two core areas of the rule of law and sustainable development. The rule of law focuses on the promotion and effectiveness of the rule of law, while sustainable development encompasses multiple aspects, including economic, political, cultural, social, and ecological environments, aiming to achieve comprehensive, coordinated, and sustainable development.

### 3.1 Evaluation indicator system for the rule of law

According to the theory of collaborative governance, three dimensions—macro institutional guarantees, meso social order, and micro practical carriers—collectively construct a three-dimensional analytical framework for social governance (41). Based on this, this article builds an indicator system for the rule of law from these three dimensions: macro institutional guarantees—rule of law environment, meso social order—social rule of law, and micro practical carriers—grassroots rule of law. These indicators, respectively, reflect the status of the macro legal environment, the overall functioning of the rule of law in society, and the implementation of grassroots legal construction. By assessing these indicators, it is possible to effectively understand the level of development of the rule of law and the existing issues, providing a basis for further enhancing the rule of law.

To measure the state of the rule of law in society, three levels can be considered: the legal environment, grassroots legal governance, and social legal governance (42, 43). These indicators reflect the legal environment established in society and the implementation status at both the overall and grassroots levels. By evaluating these indicators, one can effectively understand the level of the rule of law and identify existing issues, providing a basis for further enhancing the level of legal governance.

Therefore, to comprehensively evaluate the effectiveness of the rule of law, this paper constructs three primary indicators: rule of law environment, grassroots rule of law, social rule of law. Based on these, it further develops seven secondary indicators and eight tertiary indicators. The specific evaluation indicator system is shown in Table 1.

**Table 1:** Evaluation indicator system for the rule of law.

Level 1 indicators	Level 2 indicators	Level 3 indicators	Unit	Indicator attributes	Sign	Weight (%)
Rule of law environment	Social environment	Number of law firms	Piece	Positive	G1	10.176
		Number of lawyer staff	Person	Positive	G2	14.133
	Corporate environment	Number of permanent legal counsel units	Piece	Positive	G3	11.116
Grassroots rule of law	The number of grassroots legal mediation personnel	Number of mediators	Thousands of people	Negative	G4	19.321
	Grassroots legal mediation cases	Mediation of civil dispute cases	Piece	Negative	G5	6.846
Social rule of law	Number of cases accepted	Total number of cases accepted per 10,000 population	Pieces / ten thousand people	Negative	G6	14.697
	Number of criminal cases	The total number of criminal cases filed by the public security organs	Piece	Negative	G7	12.640
	Number of complaints	The number of appeal cases handled by the people’s Procuratorate	Piece	Negative	G8	11.072

### 3.2 Evaluation indicator system for sustainable development

Under the sustainable development indicators, the primary indicators include five aspects: economic sustainable development, political sustainable development, cultural sustainable development, social sustainable development and ecological environment sustainable development (44). These indicators reflect the sustainability status in different fields. By comprehensively assessing these indicators, one can gain a complete understanding of the overall status and existing issues of social sustainable development, providing a basis for formulating more scientific and reasonable development strategies (45).

Thus, this paper constructs primary indicators for sustainable development from five dimensions: economy, politics, culture, society, and ecological environment, and based on these, it develops 14 secondary indicators and 21 tertiary indicators. The specific evaluation indicator system is shown in Table 2.

**Table 2:** Evaluation indicator system for sustainable development.

Level 1 indicators	Level 2 indicators	Level 3 indicators	Unit	Indicator attributes	Sign	Weight (%)
Economic sustainable development	Production	GDP	100 million yuan	Positive	D1	5.731
	Income	Per capita disposable income of residents	Yuan	Positive	D2	5.386
	Consumption	Total retail sales of social consumer goods	100 million yuan	Positive	D3	4.447
		Household consumption level	Yuan	Positive	D4	5.188
Political sustainable development	Community autonomy	Subdistrict office	Piece	Positive	D5	4.225
	Rural autonomy	The number of town	Piece	Positive	D6	3.912
	Local autonomy	Number of ethnic autonomous counties level	Piece	Positive	D7	4.433
Cultural sustainable development	Education construction	Every 100,000 students in institutions of higher learning	Person	Positive	D8	10.228
	Cultural construction	The number of mass cultural service institutions	Piece	Positive	D9	2.095
		Number of art performance groups	Piece	Positive	D10	3.898
	Science and technology	Research and experimental development expenditure	100 million yuan	Positive	D11	6.648
		Number of scientific and technological achievements	Piece	Positive	D12	6.873
Social sustainable development	Social stability	Employment personnel	Thousands of people	Positive	D13	3.500
		Resident Engel coefficient	%	Negative	D14	4.272
	Infrastructure	Urban built-up area	Km^2^	Positive	D15	2.154
	Social security	Number of medical and health institutions	Piece	Positive	D16	10.248
		The inclusion of social insurance funds	100 million yuan	Positive	D17	2.945
Ecological environment sustainable development	Ecological construction	Urban green area	Ten thousand hectares	Positive	D18	2.218
		The area of national nature reserves	Ten thousand hectares	Positive	D19	7.086
	Environmental conservation	Road cleaning and cleaning area	Million square meters	Positive	D20	2.305
		Volume of household garbage clearance	Ten thousand tons	Positive	D21	2.209

## 4 Data and methodology

### 4.1 Data source

This paper empirically examines the coupling effect between the rule of law and sustainable development using panel data from China spanning the past decade, from 2014 to 2023. The relevant data are sourced from publications such as the “China Statistical Yearbook,” “China National Data,” “China Statistical Bulletin,” and databases maintained by the National Bureau of Statistics of China. To address issues of missing data for some variables, interpolation methods were employed to fill in the gaps. To ensure data validity, all numerical values in this paper have undergone standardization processing, enabling comparison and weighting among indicators with different units or magnitudes.

### 4.2 Evaluation of rule of law and sustainable development

In this paper, the entropy method is employed to calculate the levels of legal quality and sustainable development. The reason for choosing the entropy method is that it assigns weights to indicators based on the amount of information they provide, ensuring that the weighting results are authentic and reliable.

(1) Standardized treatment.

Negative indicators see Equation 1 (46):


(1) $$X_{\mathrm{ij}}=[\frac{\max(x_{1j},x_{2j},\cdots ,x_{\mathrm{nj}})-x_{\mathrm{ij}}}{\max(x_{1j},x_{2j},\cdots ,x_{\mathrm{nj}})-\min(x_{1j},x_{2j},\cdots ,x_{\mathrm{nj}})}]+0.01$$

Positive indicators see Equation 2 (47):


(2) $$X_{\mathrm{ij}}=[\frac{x_{\mathrm{ij}}-\min(x_{1j},x_{2j},\cdots ,x_{\mathrm{nj}})}{\max(x_{1j},x_{2j},\cdots ,x_{\mathrm{nj}})-\min(x_{1j},x_{2j},\cdots ,x_{\mathrm{nj}})}]+0.01$$

Where *X_ij_* is the normalized value. *maxX_ij_* Represents the maximum value in the sample evaluated by the *i* th index, and *minX_ij_* represents the minimum value in the *i* th index evaluation sample.

(2) Calculate the information entropy.

To avoid zero in the normalized value, it is necessary to translate the data. The translation formula see Equation 3 (48):


(3) $$Y_{\mathrm{ij}}=X_{\mathrm{ij}}+0.01$$

Where, *Y_ij_* represents the indicator after translation. After the translation, information entropy can be calculated, the calculation formula see Equation 4 and Equation 5 (49):


(4) $$S_{i}=\frac{-\sum_{j=1}^{n}(L_{\mathrm{ij}}\times \ln L_{\mathrm{ij}})}{\mathrm{lnn}}$$

(5) $$L_{\mathrm{ij}}=Y_{\mathrm{ij}}/\sum_{j=1}^{n}Y_{\mathrm{ij}}$$

Where *Si* is the entropy value, *L_ij_* is the proportion of item *i* index in the evaluation sample, and *n* is the number of evaluation samples.

(3) Calculate the index weight.

After obtaining the entropy value, the weight of the index can be calculated. The calculation formula see Equation 6 (50):


(6) $$W_{i}=(1-S_{i})/\sum_{j=1}^{n}(1-S_{i})$$

Where *W_i_* represents the weight of item *i* index.

(4) Calculate the comprehensive score.

After the weight is calculated, the comprehensive score of rule of law and sustainable development can be calculated separately in the *i* year, see Equation 7 (51):


(7) $$Q=\sum_{i}^{m}Y_{\mathrm{ij}}W_{i}$$

*Q* represents the comprehensive score of the national rule of law and sustainable development in year *j*, and the following is shown by *f(x)* and *g(x)*, respectively.

### 4.3 Evaluation of the coupling and coordination degree of the rule of law and the sustainable development

In this paper, on the basis of previous research, for the study of the rule of law and the sustainable development of the mutual influence state, using the coupling coordination model of the coupling degree and coupling coordination, and quantitative analysis of the interaction of the rule of law and sustainable development, reflect the coordination between the two systems and development level, the specific algorithm is as follows (52, 53):


(8) $$C=2\times {\left[\frac{f(x)\cdot g(x)}{{\left(f(x)+g(x)\right)}^{2}}\right]}^{\frac{1}{2}}$$

(9) T=αf(x)+βg(x)

(10) $$D=\sqrt{C\cdot T}$$

*C* is the coupling degree, see Equation 8; *T* is the comprehensive coordination index, see Equation 9; *D* is the coupling coordination degree, see Equation 10; *f(x)* and *g(x)* represent the comprehensive score of legal and sustainable development level respectively; the larger the value, the better the corresponding development level. *α* and *β* is the undetermined coefficient, reflecting the influence coefficient of the rule of law and sustainable development, here *α = β = 0.5*.

### 4.4 Analysis of the related factors between the rule of law and sustainable development

This paper uses the grey correlation degree model to analyze the correlation factors of the rule of law and sustainable development, so as to quantify the influencing factors affecting the development of both sides in the coupling process. When using the correlation degree model, this paper refers to Li Yiyang’s model algorithm of gray correlation degree.

(1) Step 1: define the reference sequence and compare the sequence (54), the reference sequence formula see Equation 11; The formula for the comparative sequence see Equation 12. Where i = 2013, 2014, 2015, …, 2022. a = 1, 2, 3, …, 21. b = 1, 2, 3, …, 8.

(11) $$D_{i}=(\;D_{i1},D_{i2},D_{i3}\cdots D_{i(a)}\;)\;$$

(12) $$G_{i}=(\;G_{i1},G_{i2},G_{i3}\cdots G_{i(b)}\;)$$

(2) Step 2: Use the initial value method to normalize the two groups of variables infinitely, see Equation 13 and Equation 14 (55).

(13) $$G_{i}^{\prime }(b)=G_{i(b)}/G_{i1}=(G_{i1}^{\prime },G_{i2}^{\prime },\ldots ,G_{i(b)}^{\prime })$$

(14) $$D_{i}^{\prime }(a)=D_{i(a)}/D_{i1}=(D_{i1}^{\prime },D_{i2}^{\prime },\ldots ,D_{i(a)}^{\prime })$$

(3) The third step: find the business trip sequence, maximum difference and minimum difference (56), where k = 1, 2, 3, …, n. n denotes quantity.

Differential sequences are shown in Equation 15, The maximum difference is shown in Equation 16, The minimum difference see Equation 17.


(15) $$\Delta (k)=|\;D_{i}^{\prime }(a)-G_{i}^{\prime }(b)\;|$$

(16) $$M=\max_{i}\max_{k}\Delta (k)$$

(17) $$m=\min_{i}\min_{k}\Delta (k)$$

(4) Step 4: Calculate the correlation coefficient in Equation 18. Where § is the resolution coefficient, often take § = 0.5.

(18) $$r(D_{i}(k),G_{i}(k))=(m+§M)/(\Delta (k)+§M),§\in (0,1)$$

(5) Step 5: find the correlation degree (57), see Equation 19.

(19) $$r(D_{i},G_{i})=\frac{1}{n}\sum_{\mathrm{ki}=1}^{n}r(D_{i}(k),G_{i}(k))$$

## 5 Results

### 5.1 Comprehensive development level analysis of the rule of law and sustainable development over the past decade

**Figure 3:**
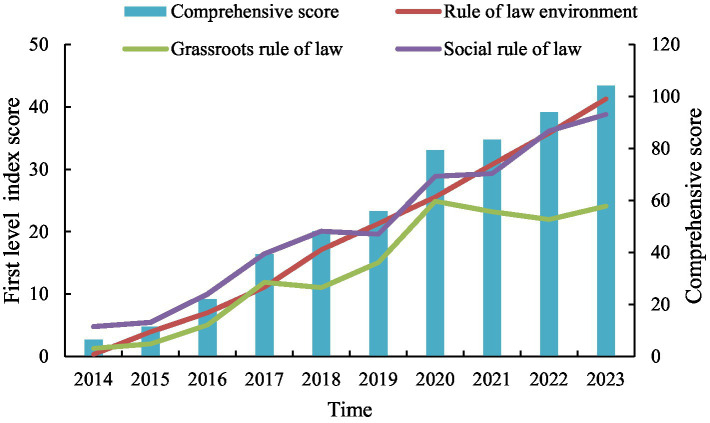
Score of the development level of the rule of law in over the past decade.

The entropy method is employed to measure the comprehensive level of the rule of law *f(x)* and the comprehensive level of sustainable development *g(x)* in China from 2014 to 2023. Figure 3 presents the comprehensive scores of the development level of the rule of law over the past decade and the development trends of the three primary indicators over the same period. Overall, China’s comprehensive level of the rule of law has consistently grown over the past decade. The comprehensive score was 6.445 in 2014 and increased to 104.163 by 2023, marking a 16-fold increase over 10 years. This substantial growth fully demonstrates the Chinese government’s high priority on the rule of law and the remarkable achievements made in this regard. When examined separately, both the rule of law environment and social rule of law have maintained rapid growth over the past decade, reflecting significant accomplishments in social legal development. At the grassroots level, there have been slight declines in some years, which are in line with the complex development situations in these areas, such as the lagged growth of the rule of law in impoverished and underdeveloped rural regions. However, the overall trend of grassroots legal development remains upward.

**Figure 4:**
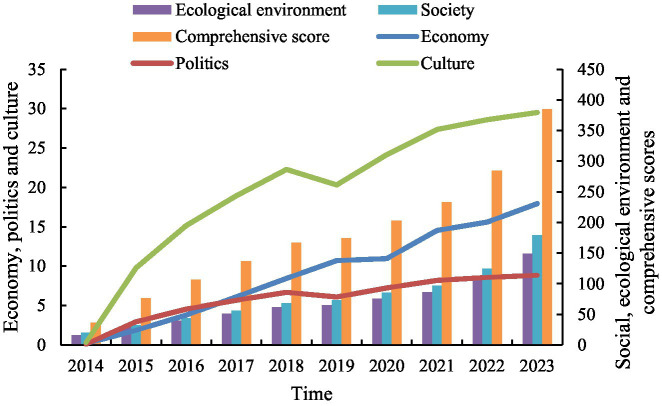
Development level score of sustainable development in over the past decade.

Figure 4 shows the comprehensive score of the development level of sustainable development in the past decade and the changes of the development level of the five first-level indicators in the past decade. On the whole, the comprehensive level of sustainable development has maintained a growth trend in the past decade. The score was 35.917 in 2014, and increased to 384.620 in 2023, an increase of 10.7 times in the 10 years, indicating that sustainable development has achieved good results. Specifically, the sustainable development of economic, political, cultural, social and ecological environment has shown an upward trend in the past 10 years, which shows that the Chinese government continuously attaches great importance to the economic, political, cultural, social and ecological environment and comprehensive scores, and promotes the sustainable development to achieve important results.

### 5.2 Analysis of the coupling and coordination degree of the rule of law and sustainable development

According to the comprehensive evaluation value of the rule of law and sustainable development calculated by entropy method, and according to the classification standard of coupling coordination level (see Table 3), the coupling coordination degree model is used to calculate the coupling coordination degree in China from 2014 to 2023 (see Table 4), and the timing evolution of the coupling coordination degree of rule of law and sustainable development is analyzed.

**Table 3:** Classification criteria of coupling coordination level.

Coordination level	Value of the coupling coordination degree, D	Coordination degree
1	[0,0.10]	Extreme Imbalance
2	[0.10,0.20]	Severe Imbalance
3	[0.20,0.30]	Moderate Imbalance
4	[0.30,0.40]	Minor Imbalance
5	[0.40,0.50]	Near Imbalance
6	[0.50,0.60]	Barely Coordinated
7	[0.60,0.70]	Primary Coordination
8	[0.70,0.80]	Intermediate Coordination
9	[0.80,0.90]	Good Coordination
10	[0.90,1]	Excellent Coordination

**Table 4:** Coupled coordination degree between the rule of law and sustainable development.

Time	Value of coupling degree, C	Coordinated index T values	Value of the coupling coordination degree, D	Coordination level	Coordination degree
2014	1	0.01	0.1	2	Severe Imbalance
2015	0.94037	0.09126	0.29295	3	Moderate Imbalance
2016	0.99380	0.18752	0.43169	5	Near Imbalance
2017	0.99722	0.31723	0.56245	6	Barely Coordinated
2018	0.99810	0.40395	0.63497	7	Primary Coordination
2019	0.99294	0.45266	0.67042	7	Primary Coordination
2020	0.97679	0.60998	0.77189	8	Intermediate Coordination
2021	0.98694	0.67227	0.81455	9	Good Coordination
2022	0.99364	0.79705	0.88993	9	Good Coordination
2023	1.00000	0.99000	0.99499	10	Excellent Coordination

**Figure 5:**
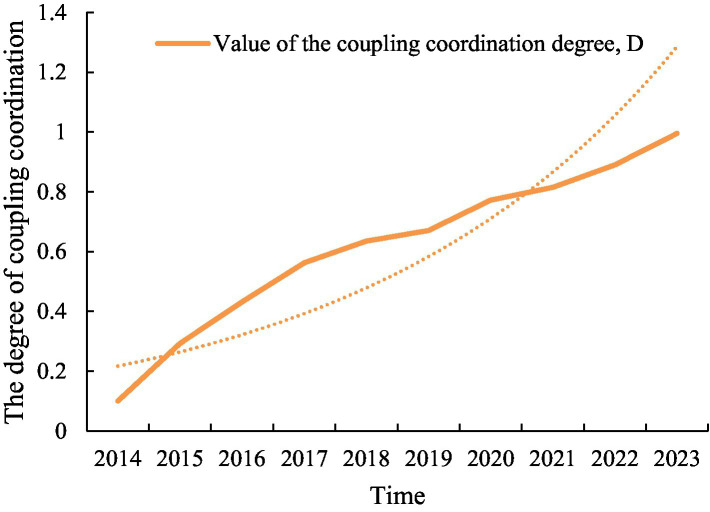
D value of coupled coordination between rule of law and sustainable development.

As a comprehensive index of the coupling coordination degree, the D value of the coupling coordination degree reflects the level of coordination development between the rule of law and sustainable development (Figure 5). As can be seen from Table 4, the coupling coordination degree between the two from 2014 to 2023 showed an obvious upward trend, from 0.10000 in 2014 to 0.99499 in 2023, which indicates that the coupling coordination development level between the rule of law and sustainable development is constantly improving. It is specifically divided into the following stages:

Low-level coordination stage (2014–2016): In this stage, the coupling coordination degree is low, and the coordination levels are severe, moderate and near misalignment. This shows that the coordinated development between the rule of law and sustainable development is still in the initial stage, and the interaction and coordination between the two have not yet formed an effective interaction.

Progressive improvement stage (2017–2020): From 2017, the degree of coupling coordination began to increase significantly, from barely coordination to intermediate coordination. At this stage, the level of coordinated development between the rule of law and sustainable development has been significantly improved, and the interaction and coordination between the two began to form an effective interaction.

High-level coordination phase (2021–2023): From 2021, the coupling coordination has increased further and remained at the level of good coordination in 2021 and 2022, and finally reached quality coordination in 2023. At this stage, the level of coordinated development between the rule of law and sustainable development has reached a high level, and the interaction and coordination between the two has formed a close interaction.

### 5.3 Analysis of the related factors between the rule of law and sustainable development

In this study, the grey correlation degree model was used to analyze the association factors affecting the rule of law and sustainable development to further quantify the influencing factors affecting the development of both parties during the coupling process. The results are shown in Table 5. The two tables show the gray correlation coefficient of the new two.

**Table 5:** The gray correlation between the rule of law and sustainable development.

Five indicators		Rule of law environment			Grassroots rule of law		Social rule of law			Average correlation degree
		G1	G2	G3	G4	G5	G6	G7	G8	
Economic sustainable development	D1	0.879883	0.920895	0.897157	0.854786	0.645086	0.880894	0.801978	0.758858	0.829942
	D2	0.877988	0.919510	0.896954	0.837761	0.659558	0.869001	0.804262	0.762068	0.828388
	D3	0.910827	0.848273	0.890623	0.880491	0.664105	0.882680	0.834596	0.725467	0.829633
	D4	0.876537	0.894170	0.893966	0.834610	0.659190	0.863785	0.800221	0.757731	0.822526
Political sustainable development	D5	0.932194	0.845346	0.922728	0.889683	0.675084	0.878844	0.819668	0.735512	0.837382
	D6	0.855036	0.803347	0.859658	0.809290	0.666250	0.806506	0.799338	0.710884	0.788789
	D7	0.679076	0.647185	0.677528	0.709223	0.550644	0.673204	0.646604	0.597434	0.647612
Cultural sustainable development	D8	0.757301	0.828216	0.766100	0.762493	0.673153	0.791149	0.744132	0.767554	0.761262
	D9	0.637876	0.695178	0.649705	0.643835	0.671005	0.647953	0.609043	0.688616	0.655401
	D10	0.503252	0.504925	0.486597	0.463707	0.499927	0.470011	0.521484	0.500140	0.493755
	D11	0.818486	0.906990	0.832150	0.813640	0.656993	0.840126	0.779013	0.779496	0.803362
	D12	0.786122	0.868997	0.799274	0.779772	0.683368	0.797838	0.750403	0.746003	0.776472
Social sustainable development	D13	0.535022	0.532900	0.519361	0.499289	0.507808	0.508446	0.551695	0.481480	0.517000
	D14	0.673456	0.649461	0.650014	0.620872	0.687114	0.630029	0.663246	0.572408	0.643325
	D15	0.919444	0.847257	0.898120	0.882973	0.678824	0.899221	0.851100	0.731905	0.838606
	D16	0.712091	0.779821	0.720654	0.716475	0.701493	0.737064	0.683739	0.741765	0.724138
	D17	0.903495	0.866648	0.903292	0.882363	0.643009	0.912025	0.827373	0.746318	0.835565
Ecological environment sustainable development	D18	0.904813	0.914003	0.911179	0.846129	0.668278	0.873564	0.814613	0.755017	0.835950
	D19	0.642310	0.624685	0.645831	0.688446	0.523696	0.653275	0.641889	0.564297	0.623054
	D20	0.899547	0.908017	0.890777	0.837139	0.678850	0.868594	0.817611	0.760228	0.832595
	D21	0.837367	0.771000	0.825417	0.836554	0.680182	0.810992	0.838002	0.686619	0.785767
Average correlation degree		0.787720	0.789373	0.787480	0.766168	0.641601	0.775962	0.742858	0.693800	

#### 5.3.1 Correlation analysis of the rule of law affecting sustainable development

According to the calculation results in Table 5, the correlation of the rule of law on sustainability is analyzed first (see Table 6). As can be seen from Table 6, the correlation degree of legal indicators to sustainable development is generally high, with the correlation coefficient both exceeding 0.6. On the whole, the rule of law environment, social rule of law and grassroots rule of law on sustainable development rank 1, 2, and 3 respectively, this shows that the rule of law environment has the highest effect on sustainable development, with the average correlation coefficient of 0.737540; the influence of social rule of law on sustainable development is second, and the average correlation coefficient is 0.737504; the lowest influence effect is the grassroots rule of law, and the average correlation coefficient is 0.703884. This highlights the focus of the current sustainable development for the rule of law, that is, to pay more attention to the role of the rule of law environment and social rule of law. Specifically, in the first-level, the correlation coefficient of G2, G1 and G3 for sustainable development ranked 1,2nd and 3rd among the eight three-level indicators, showing their main influence on sustainable development. In the social rule of law, G6 ranks fourth in eight indicators, so G6 should pay special attention to promoting sustainable development from the perspective of social rule of law. From the perspective of grassroots rule of law, G4 ranked the fifth, but it still cannot be ignored.

**Table 6:** The gray correlation degree of the rule of law on sustainable development.

Rule of law		Sustainable development	Ranking	Average score	Average ranking
Rule of law environment	G1	0.787720	2	0.788191	1
	G2	0.789373	1		
	G3	0.787480	3		
Grassroots rule of law	G4	0.766168	5	0.703884	3
	G5	0.641601	8		
Social rule of law	G6	0.775962	4	0.737540	2
	G7	0.742858	6		
	G8	0.693800	7		

#### 5.3.2 Correlation analysis of sustainable development affecting the rule of law

Based on the calculation results in Table 5, the correlation of sustainability affecting the rule of law (see Table 7). Table 7 shows the grey correlation degree coefficient of sustainable development on rule of law and its ranking. The average coefficient of correlation degree of sustainable development on rule of law is 0.748120, with strong correlation. On the whole, among the five first-level indicators under the sustainable development, the grey correlation degree of economic sustainable development, ecological environment sustainable development, political sustainable development, social sustainable development and cultural sustainable development on the rule of law is decreasing successively. Among them, the average score of economic sustainable development was 0.827622, ranking first, highlighting that economic sustainable development is the first driving force to promote the development of the rule of law. Secondly, the average correlation coefficient of ecological environment sustainable development and political sustainable development both exceeded 0.750, showing its important role in promoting the rule of law. Specifically, the top five indicators among the 21 three-level indicators are D15, D5, D18, D17 and D20, which shows the key role of the five indicators in promoting the rule of law. However, the top five three-level indicators do not involve the four three-level indicators of economic sustainable development, which indicates that they need to be systematically planned.

**Table 7:** Grey relevance of sustainable development to the rule of law.

Sustainable development		Rule of law	Ranking	Average score	Average ranking
Economic sustainable development	D1	0.829942	6	0.827622	1
	D2	0.828388	8		
	D3	0.829633	7		
	D4	0.822526	9		
Political sustainable development	D5	0.837382	2	0.757928	3
	D6	0.788789	11		
	D7	0.647612	17		
Cultural sustainable development	D8	0.761262	14	0.698051	5
	D9	0.655401	16		
	D10	0.493755	21		
	D11	0.803362	10		
	D12	0.776472	13		
Social sustainable development	D13	0.517000	20	0.711727	4
	D14	0.643325	18		
	D15	0.838606	1		
	D16	0.724138	15		
	D17	0.835565	4		
Ecological environment sustainable development	D18	0.835950	3	0.769341	2
	D19	0.623054	19		
	D20	0.832595	5		
	D21	0.785767	12		

## 6 Discussion

Based on this research, the following discussions can be made:

(1) System theory: The system theory provides a key paradigm for the analysis of the coupled and coordinated development of rule of law and sustainable development. System theory provides a critical paradigm for analyzing the coupled and coordinated development of the rule of law and sustainable development. It transcends a singular, linear perspective by viewing both as dynamic systems with structural connections. Together, they form a symbiotic network of institution-practice that empowers both directions, revealing the mutual influences between the rule of law and sustainable development. This insight holds significant theoretical value in enhancing governance effectiveness.
(2) Overall Trend of Development Levels: The comprehensive development levels of the rule of law and sustainable development show a gradually increasing trend from 2014 to 2023. Regarding the comprehensive development level of the rule of law, there has been consistent growth from 2014 to 2023, with a rapid growth rate from 2014 to 2020, followed by a slowdown from 2021 to 2023. For sustainable development, the comprehensive level has also maintained a growth trajectory, with a faster growth rate from 2019 to 2023. It is expected that both will continue to maintain this growth trend in the future.
(3) Coupling Coordination Degree: From 2014 to 2023, the coupling coordination degree between the rule of law and sustainable development shows a significant upward trend. The two have experienced a low-level coordination phase, a gradual improvement phase, and a high-level coordination phase, evolving from severe imbalance to high-quality coordination. It is anticipated that the coupling coordination mechanism will become more refined in future developments.
(4) Influencing Factors: Through gray relational analysis, this study further reveals the correlating factors between the rule of law and sustainable development. The results indicate that the relevance of the Rule of law environment, social rule of law, and Grassroots rule of law to sustainable development decreases in that order, emphasizing the importance of improving the rule of law in promoting sustainable development. At the same time, the influence of economic sustainable development, ecological environment sustainable development, political sustainable development, society sustainable development, and cultural sustainable development on the rule of law decreases in order.

## 7 Conclusion

In order to further promote the coupling and coordinated development of rule of law and sustainable development, we put forward corresponding conclusions and suggestions.

### 7.1 Macroscopic-mesoscopic-microscopic: comprehensive advancement of the rule of law in the new era

Strengthening the rule of law is a crucial means of promoting sustainable development, and this should be approached from the macroscopic, mesoscopic, and microscopic levels. First, at the macroscopic level, it is important to cultivate a robust legal environment (58). This involves not only enhancing the promotion and education of laws and regulations to improve the legal awareness and literacy of the general public but also actively advocating for the spirit of the rule of law. Citizens should be encouraged to defend their rights according to the law and to conduct affairs legally, thereby creating a strong legal atmosphere. Secondly, at the mesoscopic level, it is necessary to enhance social legal structures. This requires improving legal regulations in key areas such as environmental protection, resource management, and social responsibility and formulating more comprehensive laws. Additionally, efforts should be increased to combat illegal activities and ensure effective enforcement of the law, thereby elevating the level of legal governance in society (59). Finally, at the microscopic level, efforts should focus on strengthening grassroots legal construction. This entails actively pushing legal resources towards grassroots levels, enhancing the development of local legal institutions and personnel, and improving the professionalization of grassroots legal work. Furthermore, guidance and supervision of grassroots legal efforts should be strengthened, promoting legal participation by local communities and self-governing organizations in grassroots governance.

### 7.2 Diverse-coordinated-sustainable: the comprehensive goals of sustainable development

Economic, ecological, political, social, and cultural dimensions constitute an effective system for diversified interactive development in sustainable progress. At the economic level, it is essential to promote the transformation of economic development paradigms, shifting from a traditional model reliant on resource consumption to an innovation-driven knowledge economy. This includes encouraging enterprises to increase R&D investment (60), and drive industrial structure towards high-end, intelligent, and green development. In terms of ecological environment, strict ecological protection measures should be implemented, enhancing the protection and restoration of ecosystems, and improving their service functions. At the political level, the establishment and improvement of political policy-making procedures are necessary, including the development of a sound legal and regulatory framework to ensure that political guidance operates within the bounds of law, thus promoting the scientific and equitable implementation of policies (61). Socially, we must emphasize fairness and inclusivity, reduce the wealth gap, and enhance the education and health levels of the population, providing a solid human resource foundation for sustainable development. Culturally, investment in education must be strengthened, not only to improve the overall quality of the education system but also to elevate the overall quality of citizens and cultivate high-level talent (62). A diverse and creative array of cultural activities should be developed to enrich public life and stimulate social innovation vitality.

### 7.3 Advancing the synergy of the rule of law and sustainable development

To synergistically promote the rule of law and sustainable development, we need to adopt a series of comprehensive strategies to ensure their mutually reinforcing progress. Specifically, we should first comprehensively review and improve the existing legal and regulatory framework to ensure it fully reflects the core principles of sustainable development, timely revising or abolishing regulations that do not align with sustainable development goals. Based on this foundation, we should accelerate the formulation of a series of new regulations aimed at strengthening environmental protection, optimizing resource management, and clarifying social responsibilities, thus providing robust legal support and institutional guarantees for the practice of sustainable development.

## Data Availability

The original contributions presented in the study are included in the article/supplementary material, further inquiries can be directed to the corresponding author.
